# The Drug Habit: Its Treatment*Read at the Calvert meeting of the Brazos Valley Medical Association, May 14, 1901, and by unanimous vote ordered published.—Ed.

**Published:** 1901-11

**Authors:** M. K. Lott

**Affiliations:** Cameron, Texas


### TEXAS MEDICAL JOURNAL.

ESTABLISHED JULY, 1885.

PUBLISHED MONTHLY.—SUBSCRIPTION $1.00 A YEAR.

VOL. XVII. AUSTIN, NOVEMBER, 1901. No. 5.

### Original Contributions.

For Texas Medical Journal.

### The Drug Habit: Its Treatment.*

BY M. K. LOTT, M. D., CAMERON, TEXAS.

MR. PRESIDENT AND FELLOWS OF THE ASSOCIATION: The subject selected for your consideration is by no means new. It has engaged the careful thought of the profession many years. But not until the recent past has there been much progress in the successful management of the drug habit, even in hospitals and sanitaria and private "retreats."

I have examined the works on therapeutics at my disposal, and I have been unable to find more than a hint at the value of the different remedies which I use and will discuss in this paper. What mention I have found has been brief, and, usually, condemnatory. The statements made by most of the authorities I have consulted, are entirely at variance with my experience with the remedies referred to. I have experimented with them for the past three years, and in some twenty-five cases, and I feel that it is but right that I should give this Association and the profession generally, the benefit of that experience. If you should try the treatment I use, and which I will presently give you, and have the same results

*Read at the Calvert meeting of the Brazos Valley Medical Association, May 14, 1901, and by unanimous vote ordered published.—ED.

that I have witnessed, the gratitude of those whom you rescue from a terrible thraldom will be showered upon you.

The treatment I shall give you is identically that pursued in some private institutions by men who advertise to cure the drug habit in forty-eight hours, and who make a secret of the treatment they use, and who charge a large fee, one hundred dollars, and over. I have no fight to make on those men. If they prefer to make their profession a money-getting one, instead of a science, practiced primarily for the alleviation of human suffering, it is only to be regretted, and we cannot help it. But these institutions are for those who can pay large fees. Patients should receive better treatment and enjoy more comforts in institutions especially arranged for such cases than in private houses. But if the treatment I refer to is to be left solely in the hands of the advertising drug curers who use it solely as a money maker, what is to become of the thousands of unfortunates who have no money?

There is not an intelligent physician in Texas who cannot cure the most desperate case of morphinism; provided the patient has no organic lesion, or is not so old and emaciated that the vital powers are beyond all recuperative agencies. This he can do at home, with but little help, less expense, and without danger.

The hypodermic use of medicine has increased the number of drug habitués throughout the country, until the cure of the habit, and the restoration of the patient, is one of the most important duties of the physician; and he who understands it and practices it, is indeed a benefactor to the human race.

I wish to emphasize the fact that patients are not cured in forty-eight hours. On the contrary, it takes quite a while for the cure; they are relieved, the habit broken, and it only remains to repair the damage done to the general health. The man who has been taking morphine in considerable quantities for a long time has his vital forces very much lowered, his digestion and appetite greatly impaired, his bowels are so constipated that it is with the greatest difficulty they can be made to act. There is paralysis of the vaso-motor nerves, and it requires a variable length of time after the withdrawal of the poison before all the important functions are restored; and until then the patient has but been relieved from the drug; he has not been cured. For weeks, if not months, after the patient has ceased to use any kind of narcotic, he will still need the watchful supervision of the physician. I emphasize this fact, for the reason that those persons who advertise to cure the habit in forty-eight hours do not state the truth, and no one should be thus deceived. True, the patient, at the end of this period, or soon after, say within three or four days after the withdrawal of the morphine, feels so much relieved, and is so elated, that he is ready to pay his hundred dollars, and to sign any kind of a certificate that his deliverer wishes him to make, and it is here that the management of these institutions gets in their work; for many of those who take the treatment return to the drug, and have to undergo another treatment after its withdrawal, and this may be repeated three or four times. But each time it is easier than the first, for the reason that the victim does not wait for the habit to get such a firm hold as in the first instance. He seeks relief early, before there has been much impairment of digestive assimilation and appetite; before there has been much of the drug stored in the nervous system.

In the treatment of the habit, it makes but little difference what amount of the drug is used daily, whether it be one grain or twenty grains, the one is as difficult to overcome as the other; the difference in the economy lies in the destruction wrought by the poison, and not in the quantity of the poison, or the length of time required to relieve the patient.

I have treated people over seventy years of age, and in one case I treated a baby less than two years old. I have treated those who have used the drug for fifteen years, and those who had used it but a few months. I find that it makes but little difference in the length of time, or the amount of the antidote necessary for relief. The real difference lies in the length of time required to recover from the fearful havoc wrought in the economy by the long use of the poison.

I have confined myself largely, in speaking of the drug habit, to the drug morphine. This is because it is the one most used. It makes but little difference which one is taken, unless we except whiskey. For the cure of the whiskey habit the treatment must be somewhat different. When you begin the treatment for the whiskey habit, the bowels should be thoroughly moved, and the patient persuaded to leave off for a day or two as much of the whiskey as possible.

In the treatment for the morphine habit, or other opiates, or chloral or chloroform, no preparatory treatment is necessary. It is best to begin while the patient is well under the influence of his daily dose. He sleeps longer, and is more quiet than if treatment is deferred until about time for the patient to take his daily potion. In dealing with the whiskey habit, it is different. The treatment should be deferred until the patient has come from under its influence. In either case, a full knowledge of the condition of the heart and kidneys should be obtained. While I would not hesitate to place a patient under treatment who has heart or kidney trouble if he were otherwise in fair condition, still, I think it best to know of these conditions, and use from start to finish a fair amount of strychnia nit. 1/20 gr. hypodermatically. During the first forty-eight hours there should be no food given, or at least very little, and it should be of the lightest character, for most likely the digestive process will be suspended at this time, the food will sour and aggravate a diarrhea that will set in during this period, after the withdrawal of the morphine.

I come now to speak of the antidotes that are employed, and which are the same as are used by the various sanitaria over the country. Some of these institutions claim that their methods are a profound secret, known only to themselves. This claim I believe to be utterly false; but by this secrecy they are enabled to secure large fees from persons able to pay, while the unfortunate victims of the habit, too poor to pay the fee, must continue to suffer. Their doom is sealed. His physical suffering is not only to be considered, for instead of being regarded as a sick man, which he really is, he is generally considered a moral pervert, and his appeals for relief are treated with scorn and contempt instead of kindness.

The remedies are but few, and most, if not all of them are quite familiar to the profession. They are in almost daily use. They are: Duboisine, atropia, strychnia, and the hydrobromate of hyoscine, hyosciamine, pilocarpine. I have used all of these, but prefer the hydrobromate af hyoscine as the safest and most easily controlled. I usually begin the treatment by securing a quiet, well ventilated room with but a moderate degree of light, and so arranged that the light can be excluded, for, under the influence of hyoscine, or atropine, the pupil is widely dilated, and if there be too much light the eyes are liable to be injured. I secure a careful, competent nurse. He or she should be strong, and able to handle the patient, if necessary, for sometimes, if too much hyoscine be given, the patient will require to be protected against self-injury. The room should be down stairs preferably. The patient should be given a good bath and put to bed. I give 1/100 gr. of hydrobromate hyoscine, and after this I give the 1/200 gr. from thirty minutes to one hour for from twenty-four to forty-eight hours, until the patient has taken from forty to sixty doses, sometimes decreasing it, and sometimes omitting a dose altogether, watching carefully the pulse and respiration. The object being to secure hyoscine intoxication, which is readily recognized by the restlessness, dilated pupils, dry throat, hallucinations, etc., which it produces. When this stage is reached,

I give the drug just often enough to keep up the delirium from twenty-four to forty-eight hours.

It may be necessary during this period, to give a small dose of morphine, even one-fourth grain, once, twice or three times; it will not affect the result in the end.

Generally it is not necessary during this period to give anything else, though it may be necessary to give strychnia, or nitro-glycerine, or digitalis for the heart; but always remember that hyoscine antidotes morphine, and that morphine antidotes hyoscine. If the patient should get too much of one, I give the other.

Usually the first few hours, sometimes for four or six hours, but in some instances a much longer period, the patient sleeps. In one instance the patient slept twenty-two hours. The pupils become widely dilated, and the mouth and throat are very dry, and the patient loses the power of co-ordination and would be unable to stand, if permitted to get out of bed; he would likely fall and hurt himself. For this reason, a nurse is constantly at the bedside. Usually after a few hours sleep a delirium comes on and the patient is quite wild. He imagines that he sees and does things that do not occur. If specks are on the quilt, the patient is likely to imagine them to be chinches, and may spend hours picking them off, often remarking on their appearance and number.

During this period it is necessary to give the patient water frequently, as the throat and mouth are very dry. At the end of this period, generally, the patient is permitted to come from under any drug, and after a few hours reason is restored. The patient expresses himself as cured. He no longer craves the drug, and would not take it.

At the end of the first stage of treatment, the hyoscine, duboisin, or whichever of the group you have been using, should be left off; and begin a second stage of the treatment with pilocarpine in one-eighth grain doses, which is to be repeated every hour until its physiological effect is secured, then the time between doses is to be lengthened and kept up sufficiently often to maintain the effect, which is elimination of the drugs that have been stored in the cord and in the body; or in other words, until the last vestige of the symptoms of hyoscine have disappeared. The characteristic sneezing and attempting to yawn now commences.

During the past forty-eight hours no nourishment, or very little, has been taken, and the diarrhea has commenced. This will be likely to vex the patient for some days to come, and until now the urine has been scant and high colored.

At this period, the end of forty-eight hours, the patient has been relieved of the morphine, and thus far there has been very little or no suffering. And now pain in the knees, elbows and, the back, often sets in. Sometimes there are cramps. These are to be relieved, and here the ingenuity of the physician is called into play; for the next few days the real battle is on, and the necessities, real or imaginary, of the patient are to be met.

For the diarrhea, give sub-gallate of bismuth in 30 gr. to 60 gr. doses, with fld. extract of coto bark in 20 to 30 drop doses in water at the time of giving the bismuth. This will relieve the diarrhea in a few days. It is at this time the use of pilocarpine 1/8 gr. and strychnine 1/20 gr. or 1/30 gr., hypodermatically, should be given. Under this treatment, the dryness of the mouth and throat passes away, the urine clears up, the skin becomes moist, and the appetite begins to return, and in a week or ten days the stomach will digest almost anything you offer, and the progress is faster. But the patient is nervous and restless. He sleeps poorly, and there are the pains in the limbs and back. For these and the sleeplessness, a bath either hot or cold, or the Farradic current, will be found very beneficial. Under these methods the sleep will soon come naturally. But it may be advantageous sometimes to give large doses of the bromides of potassium, ammonium and sodium a few times, even adding chloral if necessary. But usually it is best to wait for a natural sleep. The pains can be relieved by rubbing with liniments, or by electricity, bathing, pilocarpine, strychnia, atropine, or a few doses of hyoscine.

Under the stimulus of wholesome, nutritious food, by the end of a week the patient will begin to relish these, and the countenance of the once wretched and wrecked patient, the picture of despair, feeling the warm pulses revive, with richer food and better nourishment, will regain its natural expression and color; and the brightened eye under the power of an invigorated heart, tells of the hope once more kindled in his breast.

